# *Mycobacterium smegmatis* Induces Neurite Outgrowth and Differentiation in an Autophagy-Independent Manner in PC12 and C17.2 Cells

**DOI:** 10.3389/fcimb.2018.00201

**Published:** 2018-06-19

**Authors:** Xinwei Feng, Junfeng Lu, Zitian He, Yidan Wang, Fangfang Qi, Rongbiao Pi, Ge Zhang

**Affiliations:** ^1^Department of Microbial and Biochemical Pharmacy, School of Pharmaceutical Sciences, Sun Yat-sen University, Guangzhou, China; ^2^Department of Pharmacology and Toxicology, School of Pharmaceutical Sciences, Sun Yat-sen University, Guangzhou, China; ^3^Department of Anatomy and Neurobiology, Zhongshan School of Medicine, Sun Yat-sen University, Guangzhou, China; ^4^Department of Biotechnology, School of McCormick Engineering, Northwestern University, Evanston, IL, United States

**Keywords:** *Mycobacterium smegmatis*, neurite outgrowth, differentiation, autophagy, PI3K-Akt pathway

## Abstract

Both pathogenic and non-pathogenic *Mycobacteria* can induce the differentiation of immune cells into dendritic cells (DC) or DC-like cells. In addition, pathogenic *Mycobacteria* is found to stimulate cell differentiation in the nerves system. Whether non-pathogenic *Mycobacteria* interacts with nerve cells remains unknown. In this study, we found that co-incubation with fast-growing *Mycobacteria smegmatis* induced neuron-like morphological changes of PC12 and C17.2 cells. Moreover, the *M. smegmatis* culture supernatant which was ultrafiltrated through a membrane with a 10 kDa cut-off, induced neurite outgrowth and differentiation in an autophagy-independent pathway in PC12 and C17.2 cells. Further analysis showed that IFN-γ production and activation of the PI3K-Akt signaling pathway were involved in the neural differentiation. In conclusion, our finding demonstrated that non-pathogenic *M. smegmatis* was able to promote neuronal differentiation by its extracellular proteins, which might provide a novel therapeutic strategy for the treatment of neurodegenerative disorders.

## Introduction

The microbiome has tremendous potential to influence human physiology (Sharon et al., 2014). Host-microbe interactions affect immunity, metabolism, development, and behavior (Vuong et al., 2017). Some bacteria can produce neuroactive metabolites, ranging from serotonin and γ-amino butyric acid, to dopamine and norepinephrine, to acetylcholine and histamine to influence the nervous system (Takahashi et al., 2012; Oleskin et al., 2017).

Recently, emerging researches have shown that dysbiosis of gut bacteria, commensal skin or other microbiota can affect host behavior by producing chemical signals or directly influencing host nervous systems (Ochoa-Reparaz and Kasper, 2014; Obata and Pachnis, 2016). Moreover, Microbiota have been identified within immune-privileged sites such as the central nervous system (CNS). *Proteobacteria* and *Actinobacteria* are reported to be the major commensals persistent in the human brain regardless of immune status (Branton et al., 2013).

*Mycobacterium*, a genus of *Actinobacteria*, is a highly diverse and comprises various different pathogenic and non-pathogenic species. Interestingly, although undergoing apoptotic death and necrosis, cell proliferation or differentiation was also found during the pathogenic *Mycobacterium* infection in nerves system (Rambukkana, 2010; Spanos et al., 2015). A previous study demonstrated that *M. leprae* reprogrammed Schwann cells, a type of glial cells in the peripheral nervous system, to a stage of progenitor/stem-like cells during the infection, and identified that *M. leprae*-infected Schwann cells increase cell proliferation by maintaining the infected cells in a de-differentiated state (Masaki et al., 2013). In addition, during *M. tuberculosis* infection, bacilli can be replicated within microglia, which appears to be damaged in the periphery, and could be profitable by modulating neuronal regeneration and differentiation (Spanos et al., 2015).

Additionally, environmental exposure to non-tuberculous *mycobacteria* is common. *M. vaccae*, a soil bacteria, can induce neurogenesis by stimulating the production of serotonin and norepinephrine in the brain, resulting in an antidepressant effect (Reber et al., 2016). Moreover, a polyketide toxin from *M. ulcerans* induced hypoesthesia, which leads to potassium-dependent hyperpolarization of neurons and blocking of neuronal signaling transduction, consequently annulling the pain of the lesions bacteria (Marion et al., 2014).

*Mycobacteria smegmatis* is a rapidly growing environmental species typically living in water and food sources, occasionally involving in skin or soft tissue infections. *M. smegmatis* was reported to induce the differentiation of human monocytes into mature dendritic cells directly (Martino et al., 2005). Surprisingly, we noticed that the infection of *M. smegmatis* induced neuronal morphology changes of PC12 cells. To elucidate the interactions between *Mycobacterium* and nerve cells, we investigated the morphological changes during infection and explored the mechanism of *M. smegmatis*-induced neurite outgrowth as well as neuronal differentiation.

## Materials and methods

### Cell culture

Rat adrenal pheochromocytoma cell line PC12 (Cell Bank of Chinese Academy of Sciences, Shanghai, China) were grown in RPMI 1640 (Invitrogen, USA) supplemented with 10% horse serum (HS) and 5% fetal bovine serum (FBS) (Gibco, USA). Murine neural stem cell line C17.2 (Mouse multipotent neural progenitor cells) were grown in DMEM (Invitrogen, USA) supplemented with 10% FBS. All of the cell lines were cultured in standard humidified incubators (37°C and 5% CO_2_).

### Bacterial culture

*M. smegmatis* strain ATCC 700084 was purchased from the China General Microbiological Culture Collection Center (CGMCC, Beijing, China). *M. smegmatis* were grown at 37°C for 72 h. *M. bovis BCG* and *M. tuberculosis* were grown at 37°C for 3 weeks on *Mycobacteria* L-J Culture Medium (Encode Medical Engineering Co. Ltd, Zhuhai, China) before harvesting. *E. coli* and *B. subtilis* were cultured in Luria-Bertani media at 37°C for 24 h. Heat-killed (dead) *M. smegmatis* was made by heating at 100°C for 10 min. Then, live/heat-killed *M. smegmatis* were centrifuged and suspended to 1 × 10^8^ colony-forming units (CFUs)/ml with RPMI 1640 for infection experiments.

### Conditioned medium treatment

*Mycobacteria smegmatis* were cultured in RPMI 1640 complete medium supplemented with 10% HS and 5% FBS, or in DMEM medium supplemented with 10% FBS. The RPMI 1640 or DMEM without serum was as a CM control. After *M. smegmatis* were grown at 37°C for 48 h to a final concentration of 1 × 10^9^ CFU/ml, the bacteria culture supernatant was harvested and filtered through a 0.2 μm filter membrane as conditioned medium (CM) and used for the incubation of PC12 or C17.2 cells.

### Infection experiments

Live/heat-killed *M. smegmatis* were suspended to 1 × 10^8^ CFUs/ml with RPMI 1640 medium. Then, cells were infected with bacteria at a multiplicity of infection (MOI) of 10:1. PC12 or C17.2 cells were co-cultured with *M. smegmatis* for 48 h at 37°C with 5% CO_2_.

### Differentiation induction experiment

PC12 or C17.2 cells were seeded in a 6-well plate at the density of 1 × 10^6^ cells/well and incubated for 48 h until reaching 80 % confluence. Then, cells were treated with 10% CM (v/v) from *M. smegmatis*, or 0.5 ng/ml interferon gamma (IFN-γ) (ab645, abcam, USA), or anti-INFγ (1:1,000 dilution, AF-585-NA, R&D system, USA) for 48 h. For positive control, PC12 cells were treated with 50 ng/ml nerve growth factor (NGF Sigma, USA), and C17.2 cells were treated with 50 ng/ml NGF and 50 ng/ml brain-derived neurotrophic factor (BDNF, Sigma, USA. For screening experiment of signal pathway, cells were treated with CM and different inhibitors (AG490, SP600125, U0126, SB239063, PD98059, H89, Y27632, IWP2, DAPT, BAY 11-7082, LY294002, APExBIO Technology, Houston, USA) for 48 h respectively. The morphology of the cells was observed by microscope (Nikon TE 300).

### Cell viability assay

Cell viability was measured using an MTT cell kit (Beyotime Biotechnology, Shanghai, China) following the manufacturer's instructions. Briefly, cells were seeded into 96-well plates at the density of 5 × 10^4^ cells/well and were cultured for 12 h. Then, cells were treated by CM for the indicated times. The absorbance was measured at 570 nm by an iMark Microplate Absorbance Reader.

### Neurite outgrowth measurement

PC12 or C17.2 cells were treated with CM for 48 h to analyze neurite outgrowth. Cell morphological changes were observed under a phase contrast microscope at a magnification of 200 ×. Neurite outgrowth was defined as a process with a length >2-fold of the cell body. The percentage of cells with neurite outgrowth was quantified for 300 cells/well in randomly chosen fields (*n* = 3/group). Then, the stimuli were removed, and the cells were cultured in complete cell medium for 12 h, afterwards the percentage of remaining neurite outgrowth cells was calculated again (*n* = 3/group).

### Western blotting

Total protein was extracted using a lysis buffer and protease inhibitor (Beyotime Biotechnology, China). Equivalent protein amounts were denatured in an SDS sample buffer, and then were separated by 10% SDS-PAGE and transferred onto polyvinylidene difluoride membrane. After being blocked with 5% non-fat dry milk in PBS containing 0.05% Tween-20, the blotted membranes were incubated with anti-GAP43 antibody, anti-TUBB3 antibody, anti-synaptophysin antibody (1:1,000 respectively, BS3655, BS1345, AP0013, Bioworld, China), anti-Nestin antibody, anti-MAP-2 antibody, anti-P62 antibody (1:1,000 respectively, Abcam, USA), anti-LC3 antibody (1:1,000 respectively, AF5384, AF5402, Affinity, USA) and anti-Phospho-Akt antibody, anti-Akt antibody, anti-Phospho-mTOR antibody, anti-mTOR antibody (1:1,000 respectively, 9,271, 9,272, 2,971, 2,972, Cell Signaling Technology, USA) and secondary antibody (1:5,000, Boster, China). β-tubulin protein levels were also determined by using the specific antibody (1:1,000, BS1842, Bioworld, China) as a loading control.

### Immunofluorescence staining

Cells were seeded onto glass coverslips and cultivated for 24 h until reaching 60% confluence. The medium was replaced with 1 ml of fresh complete medium and 10% CM (v/v). The cells were then cultivated for 48 h. The cells were washed with PBS three times and then were fixed with 4% paraformaldehyde. Then, the cells were incubated with primary antibodies (GAP43, synaptophysin, 1:100 respectively) at 4°C overnight. Subsequently, the cells were incubated with the FITC-conjugated secondary antibody (1:1,000, Boster, China) for 1 h at room temperature. The nuclei of cells were visualized using 4,6-diamidino-2-phenylindole (DAPI) staining. The cells were imaged at 40 × magnification using the Zeiss LSM710 confocal microscope (Zeiss, Oberkochen, Germany).

### Cytokine analysis

The quantification of cytokines in the CM was performed using a BD™ Cytometric Bead Array panel kit (BD Biosciences). The analytes included in the 6-plex kit were as follows: IL-2, IL-4, IL-6, IL-10, IFN-γ, and TNF-α. The 6 cytokines were measured by flow cytometry according to the manufacturer's instructions.

### Statistical analysis

The data was expressed as mean ± standard deviation (SD) for the indicated experiments. Paired *t-*tests were used to analyze data unless otherwise indicated. A *P* < 0.05 was considered statistically significant.

## Results

### Co-incubation with *M. smegmatis* induced neuron-like morphological changes of PC12 cells and C17.2 cells

**Figure 1:**
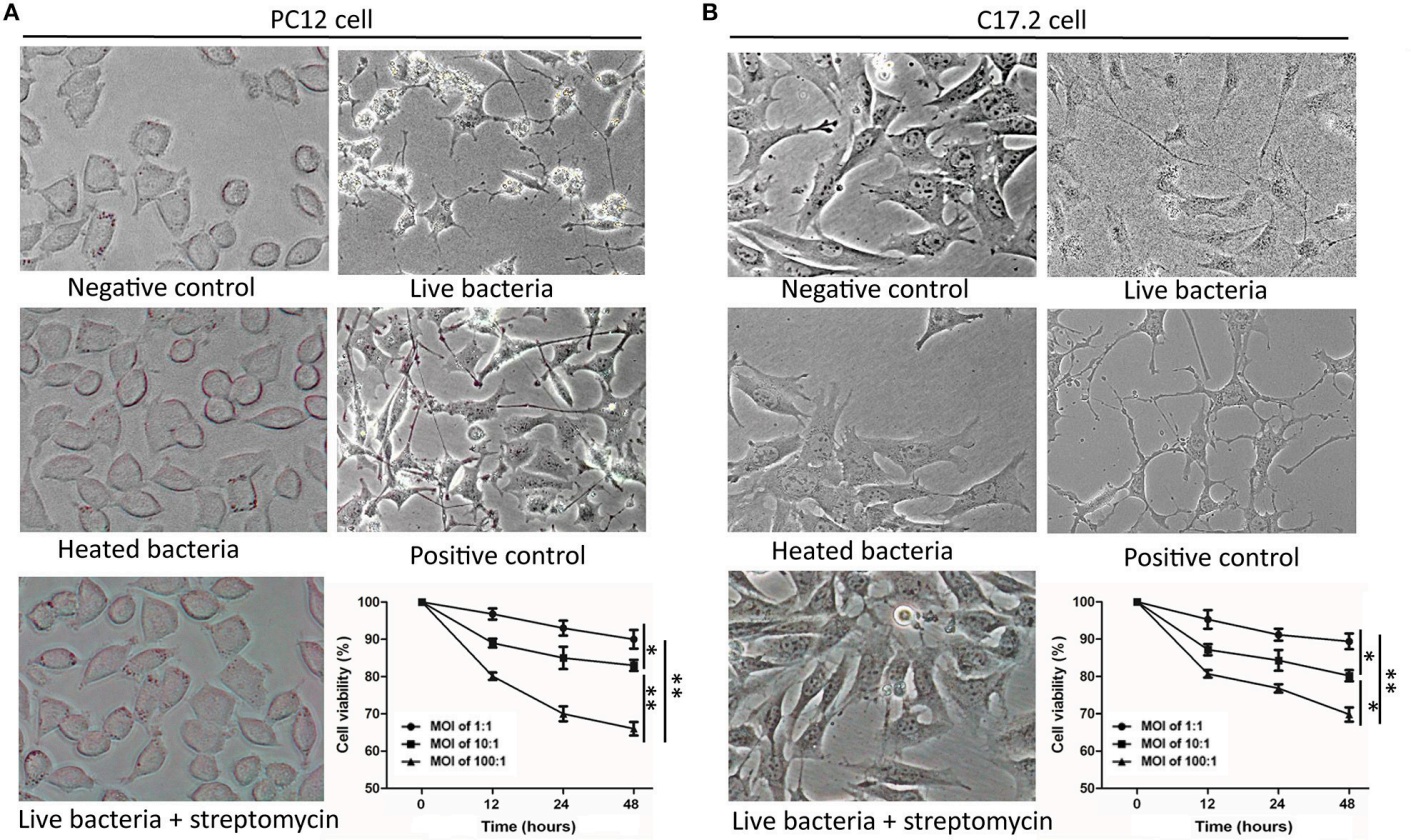
*M. smegmatis* infection induced morphological changes of PC12 and C17.2 cells. *M. smegmatis* (MOI 10:1) were added to PC12 **(A)** or C17.2 **(B)** cells for 48 h, cell morphologies were observed under different treatments (× 40). 150 ng/ml NGF was added in positive control of PC12 cells; 50 ng/ml NGF and 50 ng/ml BDNF were added to the positive control of C17.2 cells; Streptomycin (100 μg/ml) and heat-killed *M. smegmatis* (MOI 10:1) were added as dead bacteria controls. Cell viability was measured by an MTT assay with indicated infection dose and time. Quantitative results are presented as means ± S.D. for 3 replicates. ^*^P < 0.05, ^**^P < 0.01, vs. control group, *n* = 3.

To investigate whether *M. smegmatis* influences the morphology of host cells, PC12 and C17.2 cells were co-incubated with *M. smegmatis* at 37°C with 5% CO_2_ respectively. Pathogenic *M. tuberculosis* and non-pathogenic *M. bovis BCG* were used as the slow-growing *Mycobacteria* controls. As shown in Figure 1, both PC12 and C17.2 cells exhibited obvious morphological changes when co-incubated with live *M. smegmatis* compared to streptomycin-treated or heat-killed *M. smegmatis* at MOI of 10:1 (bacillui: cells) for 48 h, whereas no morphological change were observed when co-incubated with live *M. tuberculosis* or *M. bovis* BCG (Figure S1). Surprisingly, the infected PC12 and C17.2 cells showed a neuron-like morphology which is similar to NGF-differentiated PC12 or BDNF-differentiated C17.2 cells.

Moreover, MTT assays revealed that infected PC12 and C17.2 cells exhibited a reduced cell viability in a time-dependent manner and in a dose-dependent manner, but more than 80% cells were still viable at 48 h post-infection (MOI:10:1) (Figure 1). These results indicated that cells can survive under the infection condition with *M. smegmatis*, and live *M. smegmatis* further induced neuron-like morphological changes in both of non-neuronal PC12 cell line and neural progenitor/stem cells C17.2.

### Culture supernatant of *M. smegmatis* induced neuron-like morphology changes of PC12 cells

**Figure 2:**
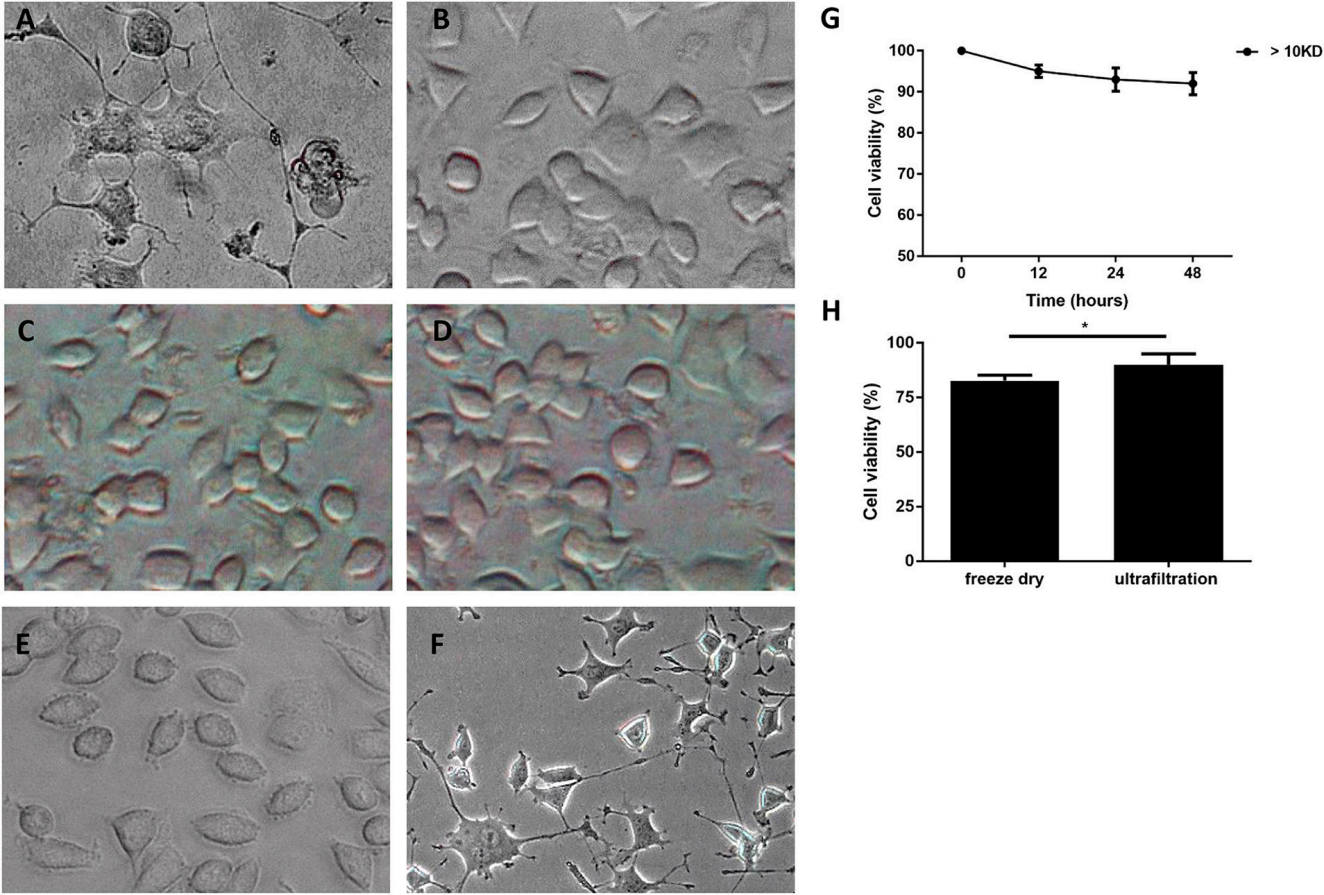
Morphology changes of PC12 cells treated with culture supernatant of *M. smegmatis*. PC12 cells treated with culture supernatant of *M. Smegmatis* **(A)**, *B. subtilis* **(B)**, *E. coli* **(C)**, and heat-killed *M. smegmatis* **(D)**; **(E,F)** < 10 kD and > 10 kD subgroup of live *M. smegmatis* culture supernatant for 48 h, and cells were observed under phase-contrast microscope (× 40). **(G)** Cell viability of PC12 treated with ultrafiltrated supernatant at indicated time; **(H)** The relative viability of cells treated by CM with freeze drying or ultrafiltration for 48 h. Quantitative results were presented as means ± S.D. for 3 replicates. ^*^P < 0.05, vs. control group, *n* = 3.

Furthermore, *M. smegmatis* was observed to proliferate well in the complete cell culture medium (including 10% FBS in RPMI 1640), whereas cannot grow in serum-free cell medium. *M. smegmatis* was then cultured for 48 h in the culture medium with 10% FBS, and the culture supernatant was collected as a conditioned medium (CM). Gram positive *Bacillus subtilis* and Gram negative *Escherichia coli* were cultured in the same culture medium and the supernatant was used as non-*Mycobacterium* bacteria controls. We observed the neurite-like initiations at 12 h, and its enhanced extensions from 24 to 48 h after treating the PC12 cells with CMs from *M. smegmatis* (Figure S2). Figures 2A–D showed that CM induced obviously neuron-like morphological changes in PC12 cells at 48 h, whereas heated supernatant and CM controls from *B. subtilis* or *E. coli* failed to induce visible changes.

Next, CM was concentrated 5-fold and preliminarily purified by ultrafiltration with the cut-off limit of 10 kD. The PC12 cells treated with the ultrafiltrated CM exhibited neuron-like changes, whereas outflow from ultrafiltration membrane did not show any gross morphological alterations (Figures 2E,F). MTT analysis showed that PC12 cells were preserved more than 90% viability when treated with the ultrafiltrated CM for 48 h (Figure 2G). However, when CM was concentrated about 5-fold by freeze drying, PC12 cells showed a significantly reduced cell viability (Figure 2H).

These results indicated that the culture supernatant of *M. smegmatis*, but not the bacteria itself, plays a role in the progress of morphological changes, suggesting that *M. smegmatis* could secrete heat-labile extracellular protein with a molecular weight >10 kD which induced the neuron-like changes.

### Culture supernatant of *M. smegmatis* induced neuronal differentiation of PC12 cells and C17.2 cells

**Figure 3:**
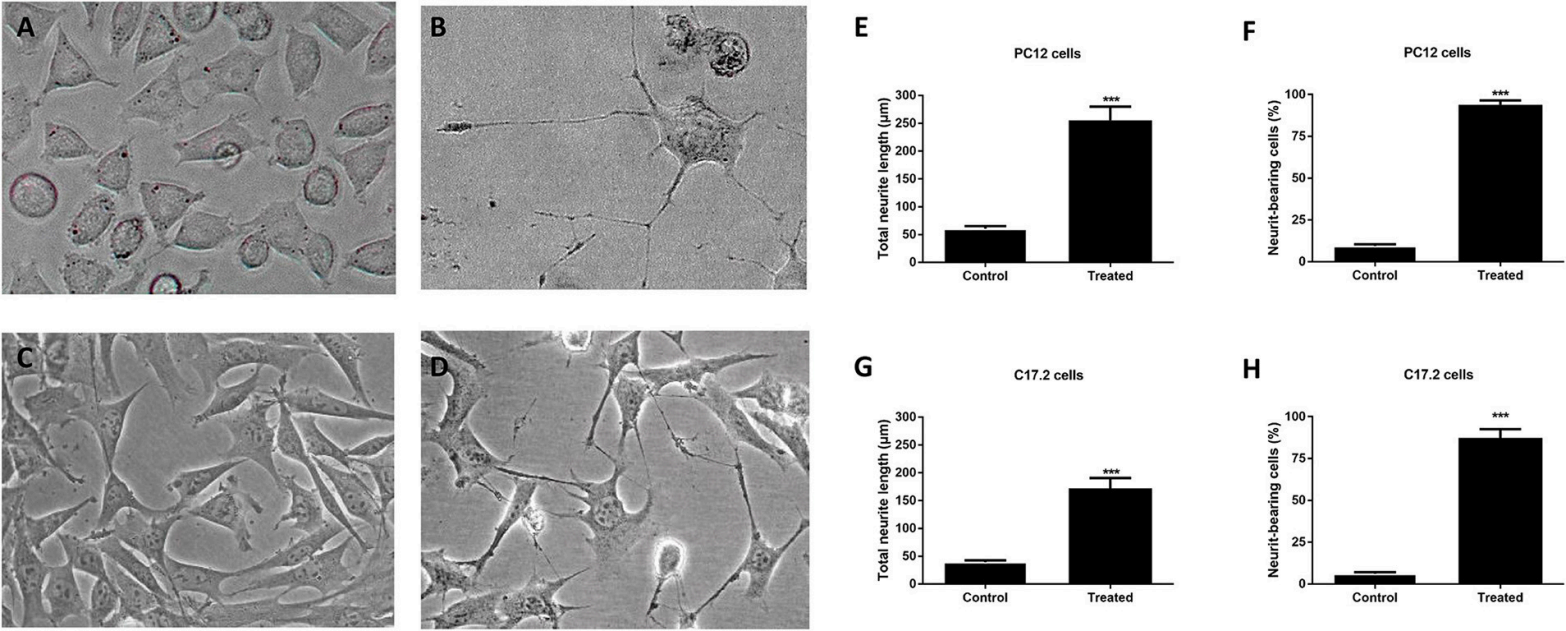
Neurite outgrowth of PC12 cells and C17.2 cells treated with culture supernatant of *M. smegmatis*. Changes of neurite length in PC12 and C17.2 cells were observed after being stimulated with *M. smegmatis* culture supernatant for 48 h. Cell morphologies of PC12 cells **(A,B)** and C17.2 cells **(C,D)** treated with *M. smegmatis* culture supernatant were observed respectively (× 40); Neurite length and neurite-bearing cells of PC12 cells **(E,F)** and C17.2 cells **(G,H)** treated with *M. smegmatis* culture supernatant respectively. Quantitative results are presented as means ± S.D. for 3 replicates. ^***^P < 0.001, vs. control group, *n* = 3.

To further elucidate whether the extracellular protein of *M. smegmatis* induced neuronal differentiation, neurite outgrowth was measured at 2 days post-infection. As shown in Figures 3A,B, a higher frequency of longer neurites as well as a higher average of neurite length were observed in *M. smegmatis*-CM-stimulated PC12 cell, and about a 5-fold increase in neurite length and an 8-fold increase in neurite-bearing cells were determined compared to untreated cells (Figures 3E,F). Similar results were also observed in CM-stimulated C17.2 cells (Figures 3C,D,G,H), suggesting that extracellular proteins of *M. smegmatis* strongly increased neurite outgrowth in PC12 and C17.2 cells.

**Figure 4:**
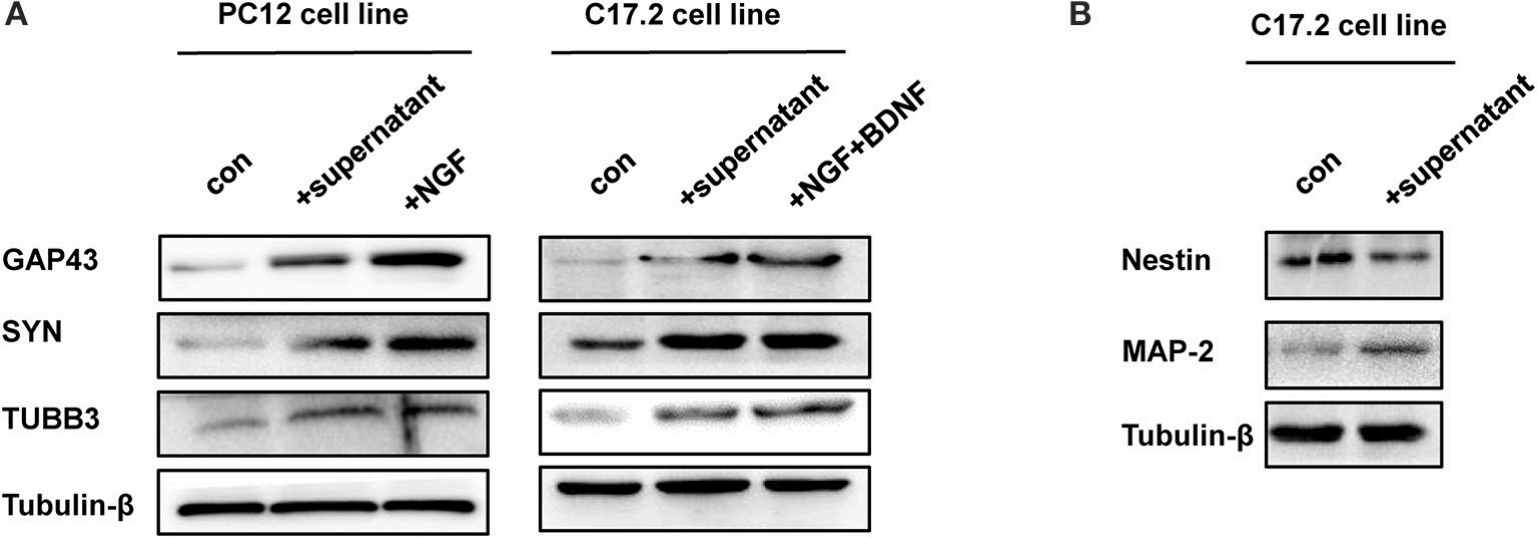
Western blotting analysis of differentiation-related markers of PC12 cells and C17.2 cells treated with culture supernatant of *M. smegmatis*. **(A)** PC12 cells and C17.2 cells were treated with/without culture supernatant of *M. smegmatis*, differentiation related markers GAP43 (Growth Associated Protein 43), SYN (Synaptophysin) and TUBB3 (Tubulin beta-3 chain) were determined by western blot analysis; **(B)** Neuron related markers Nestin and MAP-2 were determined by western blot analysis in C17.2 cells.

**Figure 5:**
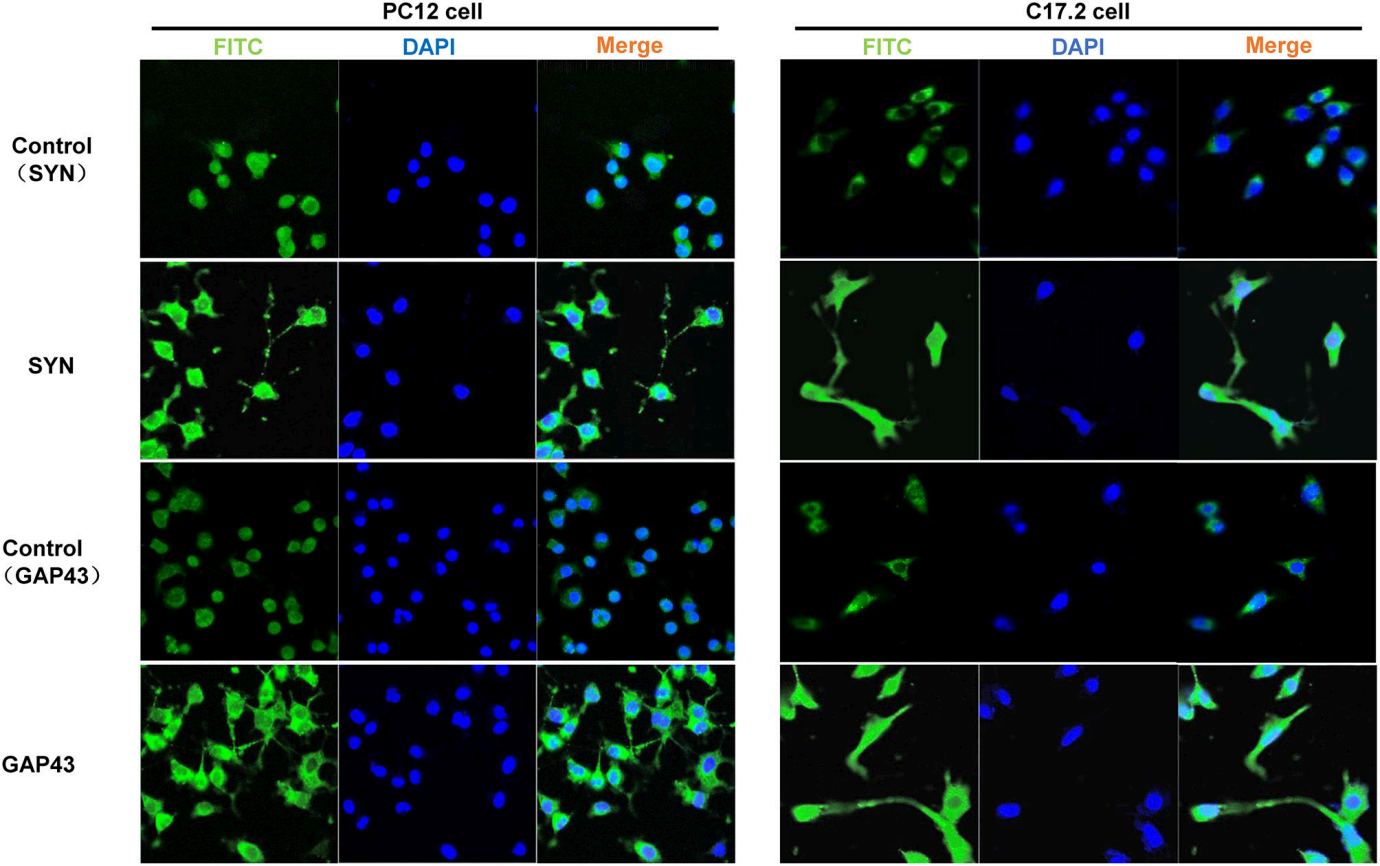
Immunofluorescence detection of differentiation markers in PC12 cells and C17.2 cells treated with culture supernatant of *M. smegmatis*. PC12 cells and C17.2 cells were treated with 10% *M. smegmatis* culture supernatant for 48 h Cells were immunofluorescence stained with GAP43 and SYN. Nuclear counterstaining was done with DAPI. Fluorescence was visualized with a laser-scanning confocal microscope (40 ×).

Additionally, three neural differentiation markers were assessed in CM-stimulated PC12 and C17.2 cells by western blotting and immunofluorescences staining respectively. As shown in Figure 4A, the expression of synaptic markers (GAP43 and SYN) and immature neuronal marker TUBB3 was up-regulated in both of CM-stimulated PC12 and C17.2 cells. In addition, the expression of mature neuronal marker MAP2 was up-regulated, whereas neural stem cell marker Nestin was down-regulated in CM-stimulated C17.2 cells (Figure 4B). Consistent with results of western blotting in Figure 4, immunofluorescence assays indicated that CM-stimulated PC12 and C17.2 cells exhibited neuron-like morphological changes with strong expression of GAP43 and SYN (Figure 5). These results further identified that neural differentiation of PC12 and C17.2 cells may be induced by the extracellular protein secreted from *M. smegmatis*.

### Culture supernatant of *M. smegmatis* induced neuronal differentiation in autophagy-independent pathways

**Figure 6:**
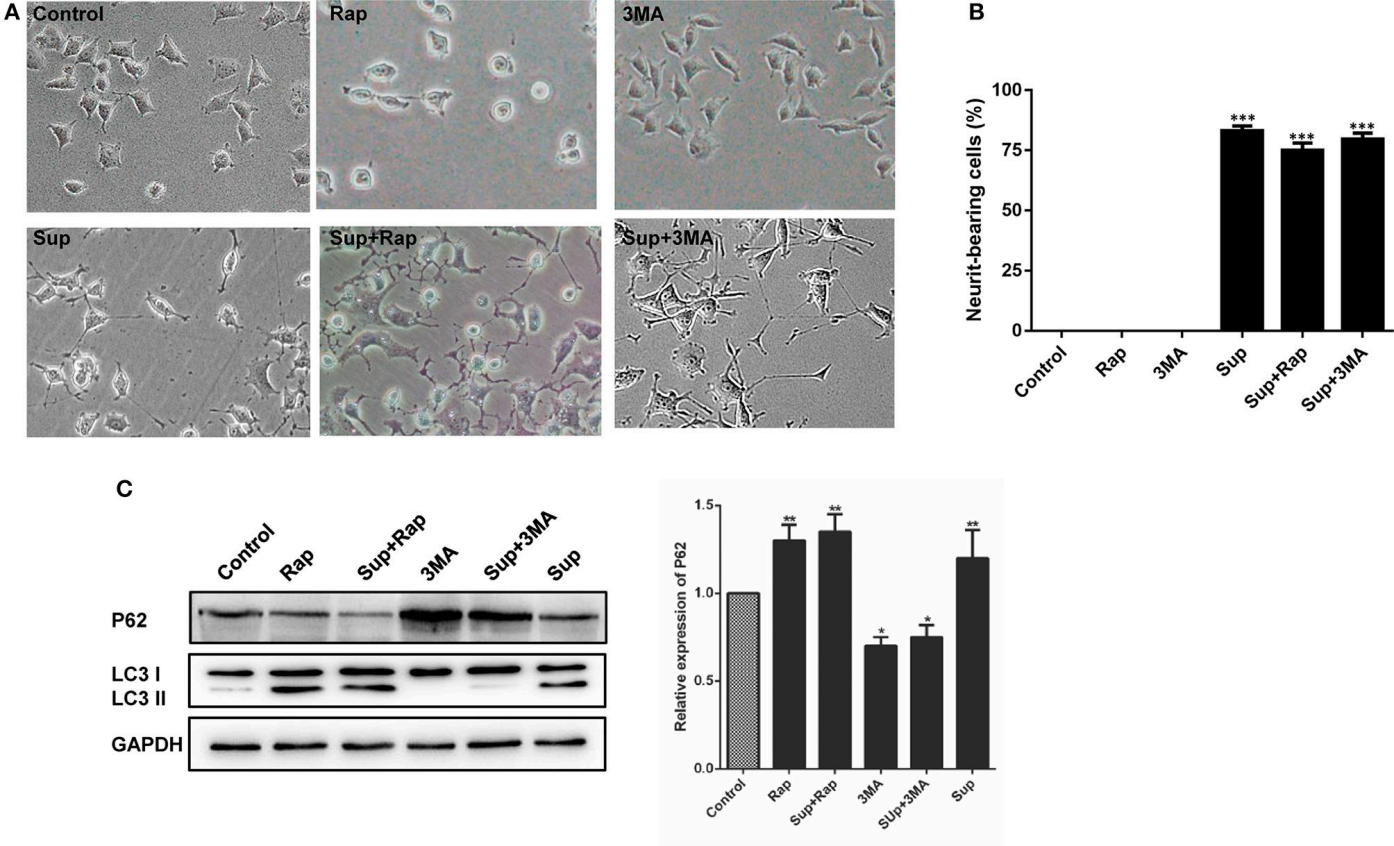
Autophagy did not affect neurite outgrowth in PC12 cells. **(A)** The expression of the autophagy markers P62 and LC3 were determined by western blot analysis in PC12 cells; **(B)** PC12 cells morphology changed and **(C)** neurite-bearing cells counted after treatment with *M. smegmatis* culture supernatant (Sup), the autophagy inducer rapamycin (1 μM, Rap), the autophagy inhibitor 3-MA (10 mM, 3-MA), Sup + Rap, and Sup + 3-MA. (20 ×) for 48 h. Quantitative results were presented as means ± S.D. for 3 replicates. ^*^P < 0.05, ^**^P < 0.01, ^***^P < 0.001 vs. control group, *n* = 3.

Autophagy is involved in the host immunity against bacterial infection. To investigate whether autophagy plays a role in CM-stimulated neural differentiation, the autophagy related proteins are further detected by Western bolt assay. As shown in Figure 6A, PC12 cells treated with CM obviously induced LC3 I to II conversion, and autophagy inhibitor 3-methyladenine (3-MA) markedly inhibited the CM-induced conversion. Interestingly, CM and autophagy activator rapamycin exhibited similar ability to induce LC3 I to II conversion. In addition, although no obvious difference was detected in the levels of autophagy receptor p62 between untreated and CM-treated PC12 cells, rapamycin treatment led to a reduced level of p62 in CM-stimulated PC12 cells. These results suggested that culture supernatant of *M. smegmatis* induced autophagy.

Additionally, the neurite-bearing cells were counted in the treated PC12 cells. As shown in Figures 6B,C, there was no obvious change in neurite outgrowth of PC12 cells neither treated with autophagy activator rapamycin or inhibitor 3-methyladenine (3-MA). The CM-stimulated neural differentiation of PC12 cannot be further affected by autophagy activator or inhibitor. Taken together, these results indicated that culture supernatant of *M. smegmatis* promoted neuronal differentiation in an autophagy-independent pathway.

### PI3K-Akt pathway is involved in the role of *M. smegmatis* induced neuronal differentiation

**Figure 7:**
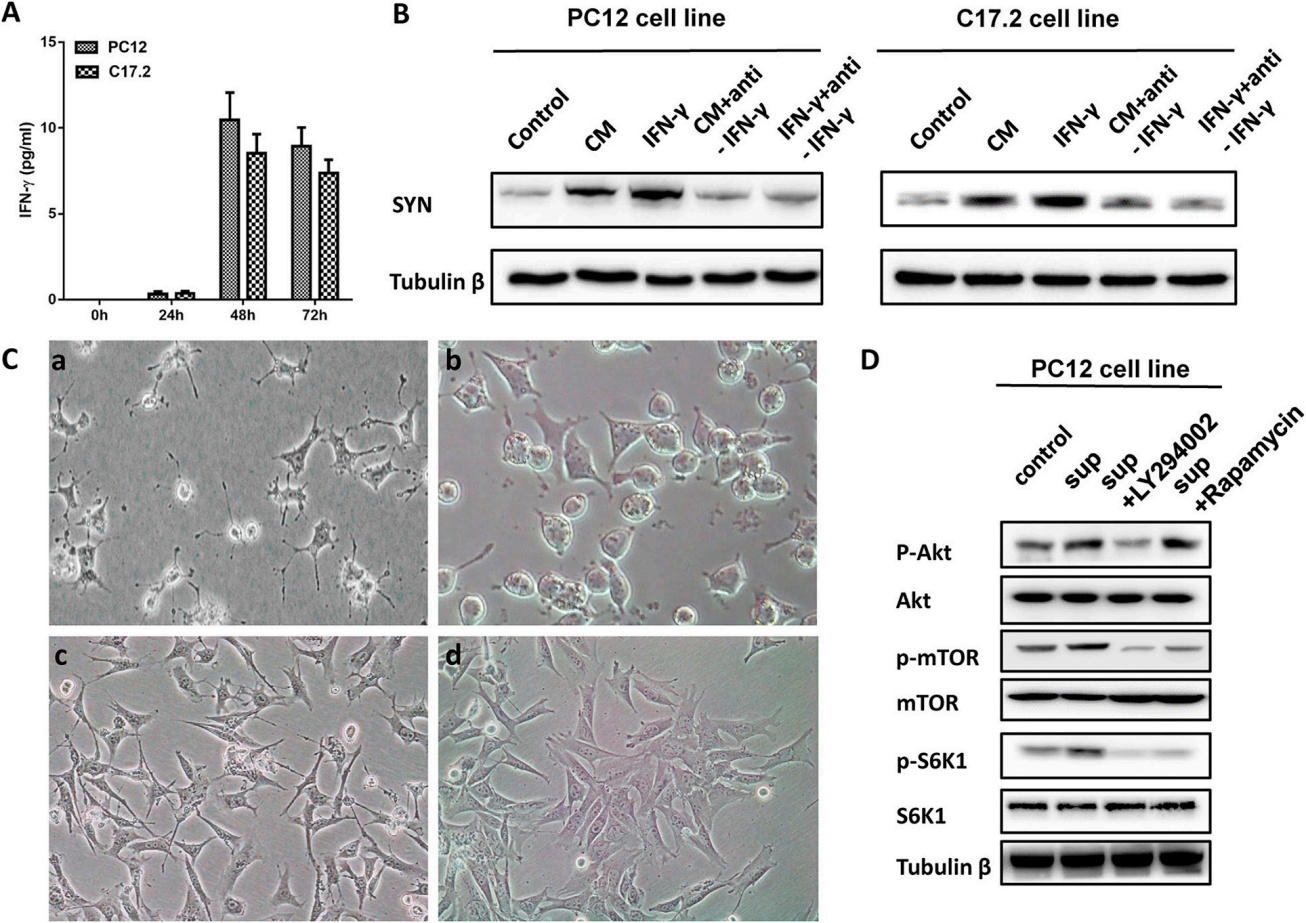
IFN-γ involved in the role of *M. smegmatis* induced neuronal differentiation of PC12 cells and C17.2 cells. **(A)** IFN-γ in the supernatant of *M. smegmatis* was measured using a cytokine bead array by flow cytometry; **(B)** Western blotting analysis of exogenous IFN-γ treatment in PC12 and C17.2 cells; **(C)** PC12 cells were treated with culture supernatant of *M. smegmatis* **(a)** and supernatant plus LY294002 **(b)** for 48 h; C17.2 cells were treated with culture supernatant of *M. smegmatis* **(c)** and supernatant plus LY294002 **(d)** for 48 h (20 ×); **(D)** PC12 cells were exposed to RMPI 1640, supernatant of *M. smegmatis*, LY29402 for 24 h respectively. Levels of p-AKT/AKT, p-mTOR/mTOR and p-S6K1/S6K1 were measured by western blot assay.

IFN-γ is a key immune mediator during mycobacterial infections, and may serve as a common mechanism of differentiation in nervous system (Kim et al., 2007). Therefore, cytokines were measured by flow cytometry using a cytokine bead array. As shown in Figure 7A, IFN-γ was detected in the supernatants of PC12 and C17.2 cells treated with CM, while the TNF-α, IL-2, IL-4, and IL-6 concentrations were below the limits of detection until 48 h. In addition, IFN-γ levels peaked (10.47 ± 1.34; 8.52 ± 0.65 pg/ml) at 48 h post-incubation and substantially decreased. Moreover, exogenous IFN-γ treatment promoted neuronal differentiation and a neutralizing antibody of IFN-γ significantly inhibited either IFN-γ or CM-induced differentiation of PC12 and C17.2 cells (Figure 7B).

Then, we screened the signal pathway which might be involved in the differentiation phenomenon through inhibitors of different pathway. Figure S3A showed that PI3K inhibitor LY294002 inhibited the cell differentiation caused by culture supernatant of *M. smegmatis*, and this result was further confirmed by the western blotting (Figure S3B).

IFN-γ is an important mediator of inflammation and may activate PI3K-Akt signaling pathways (Nguyen et al., 2001). Next, PC12, and C17.2 cells were further treated with PI3K inhibitor LY294002. As shown in Figure 7C, the CM-induced neurite outgrowth in PC12 and C17.2 was obviously inhibited by LY294002. Additionally, after PC12 cells co-cultured with the CM of *M. smegmatis* for 48 h, Western blot analysis showed that CM increased phosphorylation of Akt, mTOR and S6K1, while LY294002 inhibited the effects of p-Akt, p-mTOR, and p-S6K1 (Figure 7D).

The data indicated that *M. smegmatis* induced the production of proinflammatory cytokines IFN-γ, and IFN-γ might be involved in neuronal differentiation through the activation of the PI3K-Akt signaling pathway.

## Discussion

In this study, we demonstrated an interesting phenomenon that *M. smegmati*s infection could induce neuron-like morphological changes of PC12 and C17.2 cells. Some microbial and its metabolite can induce neurite outgrowth (Nakashima et al., 2002). Various short-chain fatty acids such as butyrate, induce morphological and biochemical differentiation of glioma or neuroblastoma toward a neuronal phenotype (Suzuki-Mizushima et al., 2002). Similar results were found in adenosine analog, retinoic acid (Tonge and Andrews, 2010). NO is reported to involve in NGF-induced differentiation of PC12 cells (Oh et al., 2010). In addition, some proteins such as cholera toxin from *Vibrio cholerae*, p15 protein from *Aspergillus oryzae*, can induce neurite outgrowth and differentiation in PC12 cells (Glineur and Locht, 1994); Skp, a 17-kDa *Escherichia coli* molecular chaperone protein, was reported to convert pluripotent P19 cells into functionally active neurons (Halder et al., 2015). In our study, the culture supernatant from the complete medium of fast-growing *M. smegmatis* but not slow-growing *M. tuberculosis* or *M. bovis* induced neuronal differentiation. Furthermore, the culture supernatant concentrated by ultrafiltration (10 kD) exhibited good activity and was inactivated when heated at 100°C. Although we couldn't separate and purify the active substances, these results suggested that the effective substances were heat-sensitive extracellular proteins, which will be secreted from fast-growing *Mycobacterium* with a sufficient concentration to induce neuronal differentiation during the infection in cultured cells

An in-depth study of neural dendrite formation is of great significance both in theories and applications. Interestingly, *M. smegmatis* can cross the modal blood–brain barrier, although the median invasion fraction for *M. smegmatis* was significantly lower than that for *M. tuberculosis* (Jain et al., 2006). In general, CNS infection can disrupt the survival, proliferation, and maturation of neural stem precursor cells (NSPC), which ultimately impair neurogenesis. However, the adult mammalian brain creates new neurons from pools of stem cells. Recent studies have shown that neurogenesis increases in response to brain injuries, neurodegenerative conditions and infection. For example, although neuronal damage in the hippocampal formation is a common feature of *S. pneumoniae* meningitis, an increased neurogenesis was found after experimental meningitis (Lian et al., 2016). Zika Virus (ZIKV)-infected cranial neural crest cells (CNCCs) undergo limited apoptosis but secrete cytokines that promote death and drive aberrant differentiation of neural progenitor cultures (Bayless et al., 2016).

The neural and the immune systems share many of the same regulatory factors such as IL-6, IFN-γ, and TNF-α (Thomson et al., 2014). The PI3K-Akt pathway that is stimulated by cytokines, and by its strong links to cell proliferation, survival and inflammation, is one of the most important signaling pathways involved in the regulation of neural functions (Yin et al., 2017; Zhang et al., 2017). Notably, IFN-γ plays an especially important role in the innate host response to microbial infections (Warren et al., 2017). Treatment with IFN-γ resulted in the increased killing of intracellular bacteria. IFN-γ also exerts both protective and pathological effects on other CNS diseases. IFN-γ promotes neuronal differentiation of some cells, such as HUCB-derived progenitors (Arien-Zakay et al., 2009). Some studies have shown that anti-viral immunity protects the NSPC population during a neonatal viral CNS infection by IFN-γ (Fantetti et al., 2016). Our study further demonstrated that IFN-γ displayed the role of neuronal differentiation during *M. smegmatis* stimulation by PI3K-Akt signaling pathways. In line with our study, IFN-γ was reported to facilitate NGF-induced neuronal differentiation in PC12 cells (Improta et al., 1988), and to regulate proliferation and neuronal differentiation by STAT1 in normal adult brains (Pereira et al., 2015). Furthermore, a recent study has reported that neonatal BCG vaccination and BCG-serum promote hippocampal neurogenesis and cognitive function in early life, accompanied with elevated IFN-γ levels both in the periphery and the brain (Yang et al., 2016).

Moreover, multiple studies have indicated a role of autophagy in neuronal differentiation (Li et al., 2014; Mirzaa et al., 2016; Fidaleo et al., 2017). Autophagy can cause neuronal differentiation (Xu et al., 2015). In our study, the culture supernatant of *M. smegmatis* promoted autophagy signaling. Besides, it is well-known starvation could induce cell autophagy, but starvation cannot induce the differentiation in our study. This results further suggested that neuronal differentiation was induced in an autophagy-independent pathway. Moreover, autophagosomes are formed in response to a number of environmental stimuli, and IFN-γ has been shown to induce autophagy. Moreover, autophagy can regulate the production and secretion of cytokines, including IFN-γ (Harris et al., 2017). Induction of autophagy has been proposed as a reasonable strategy to help neurons clear abnormal protein aggregates and survive (Heras-Sandoval et al., 2014).

*M. smegmatis* is a rapidly growing environmental species that is present in skin. Although little is known about skin microbiota, this may be the next frontier in elucidating molecular mechanisms of pathogenesis and symbiosis. The skin participates in the stress response by a local hypothalamic-pituitary-adrenal axis (HPA), peripheral nerve endings and skin cells. Pro-inflammatory cytokines and neurogenic inflammatory pathways involve in the brain-skin connection (Chen and Lyga, 2014). *M. smegmatis* and *M. tuberculosis* share many features and identical genomic sequences are a potential candidate for developing new tuberculosis vaccines (Nguyen Thi et al., 2010). It is reported that fatal disseminated *M. smegmatis* infection in a child with inherited IFN-γ receptor deficiency (Pierre-Audigier et al., 1997). In addition, administration of live *M. smegmatis* was administered to mice to produce Th1-type cellular responses (IFN-γ and IL-2) and anti-tumor effects (Young et al., 2004). These suggested the non-pathogen *Mycobacterium* could be used to promote neuronal differentiation in neurodegenerative disorders.

In summary, *M. smegmatis* infection can induce neuronal differentiation in an autophagy-independent manner *in vitro*. Although the details of molecular mechanisms of the effects remain to be elucidated, our findings suggest that live non-pathogenic *Mycobacterium* infections might not only play the role against intracellular infections, but also prompt neuronal differentiation. In particular, our findings may shed new light on the roles of non-pathogen *Mycobacterium* such as *M. smegmatis* in CNS, and may open a novel approach to treating brain tumors or neurodegenerative diseases such as Alzheimer's and Parkinson's diseases. Further studies are going to be conducted *in vivo* to investigate the effects of live *M. smegmatis* infections in mice brain and AD transgenic mice.

## Author contributions

GZ, RP, and XF contributed to the conception and design of the study. GZ and XF contributed to data acquisition, data analysis and manuscript writing. JL, ZH, YW, and FQ participated in data analysis and manuscript writing. All authors read and approved the final manuscript.

### Conflict of interest statement

The authors declare that the research was conducted in the absence of any commercial or financial relationships that could be construed as a potential conflict of interest.
