# Supplementary material for: Mycobacterium smegmatis Induces Neurite Outgrowth and Differentiation in an Autophagy-Independent Manner in PC12 and C17.2 Cells
Source: Front Cell Infect Microbiol. 2018 Jun 19;8:201. doi: 10.3389/fcimb.2018.00201 (PMC6024096; doi:10.3389/fcimb.2018.00201)
Supplement: Supplementary file 1 [file Image_1.PDF]

Fig. S 1

PC12 cell

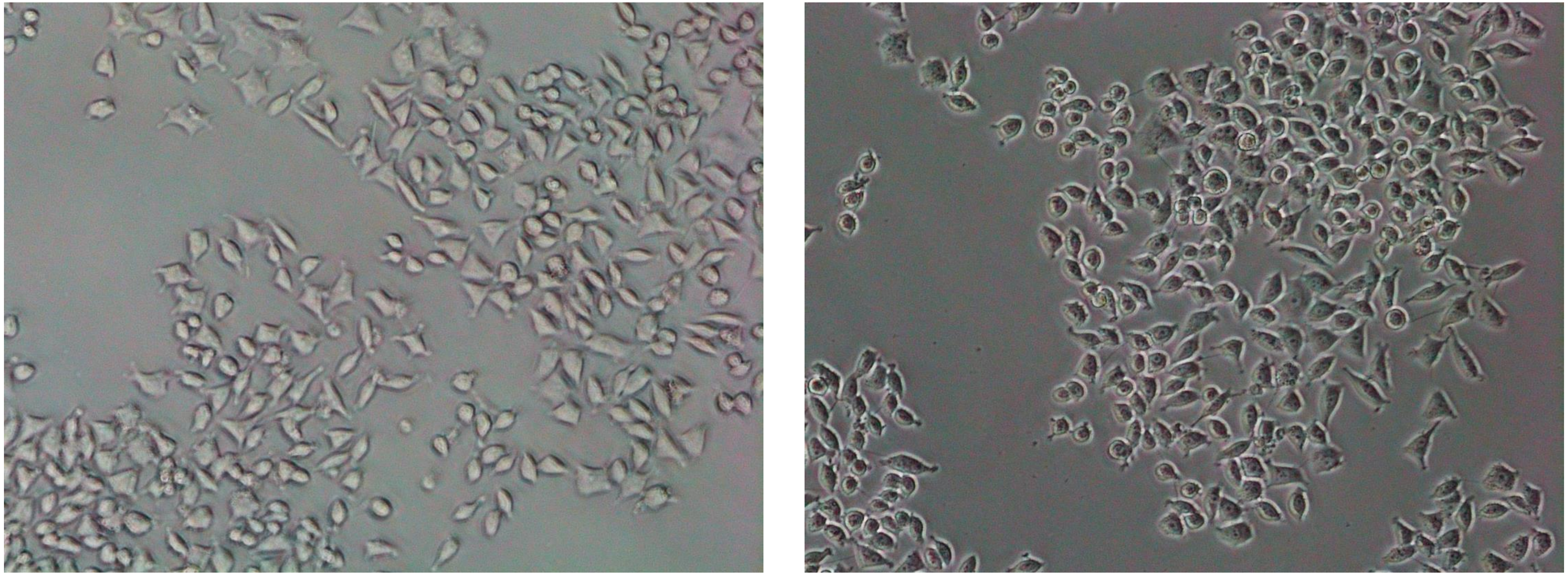

M. tuberculosis ***M. bovis***

Figure S1. Slow-growing *Mycobacteria* co-cultured with PC12 and C17.2 cells.

(A) *M. tuberculosis* and (B) *M. bovis BCG* (MOI 10:1) were co-cultured with PC12 cells for 48 h. Cell morphologies were observed under light microscope (×40).

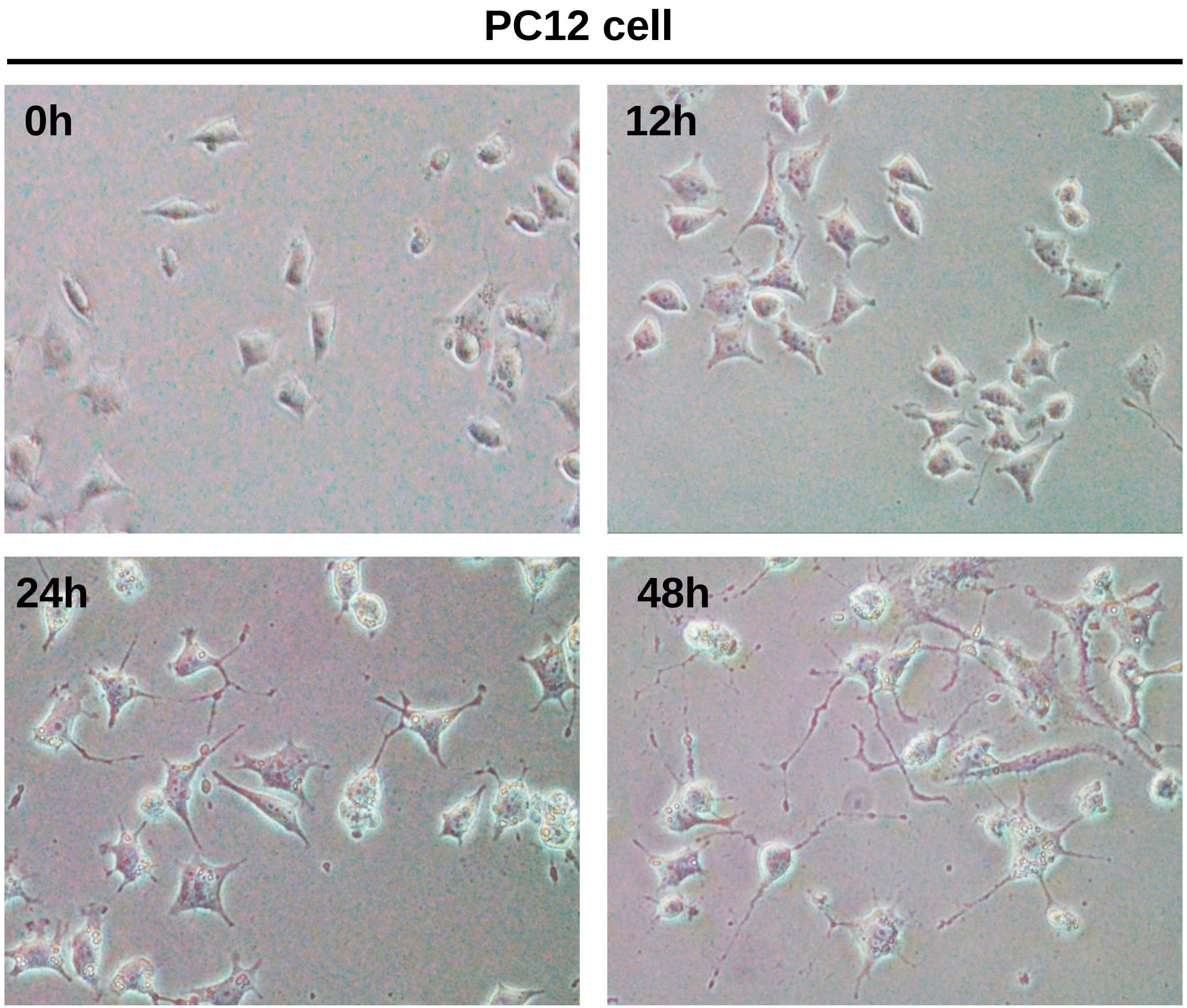

Figure S2. CM-induced neurite-like outgrowth in PC12 cells at indicated time.

CM-induced neurite-like outgrowth in PC12 cells at 0 h (A); 12 h (B), 24 h(C), 48 h(D).

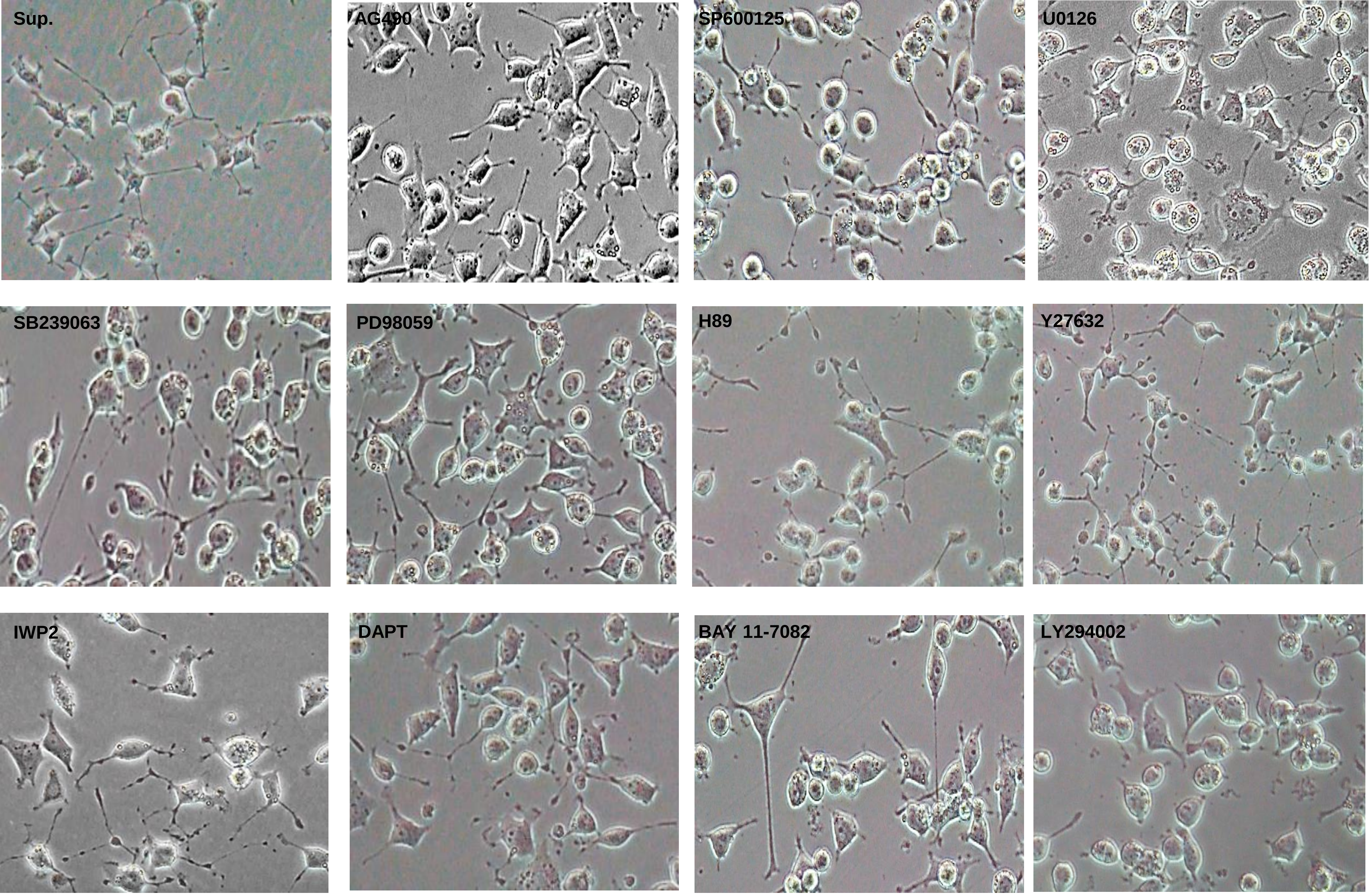
Sup.
AG490
SP600125
U0126
SB239063
PD98059
H89
Y27632
IWP2
DAPT
BAY 11-7082
LY294002

Fig. S 3A

Fig. S 3B

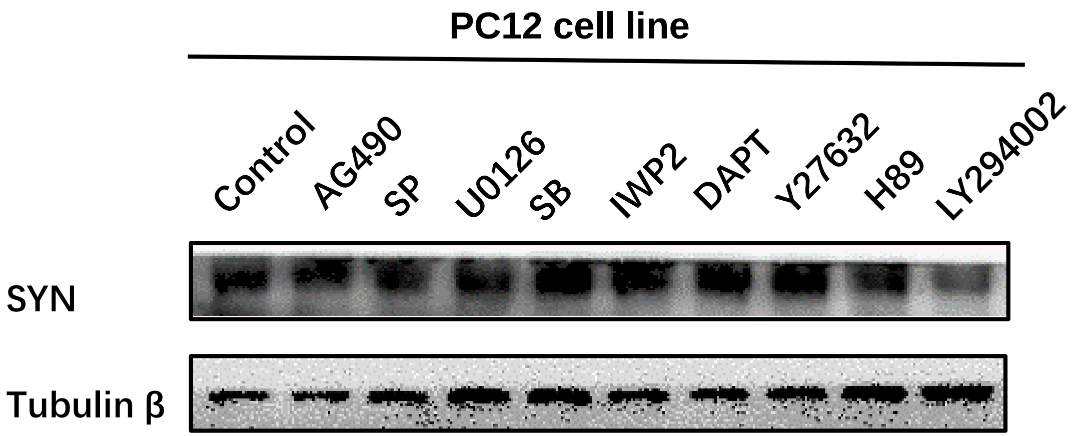

Figure S3. Screening of signaling pathways through different inhibitors. (A) PC12 cells added with culture supernatant of *M. smegmatis* and different inhibitors for 48h; (B) Western blotting analysis of the expression levels of the differentiation markers SYN in PC12 cells treated with different inhibitors. Positive: co-cultured with *M.smegmatis* culture supernatant.
